# Microfabric Vessels for Embryoid Body Formation and Rapid Differentiation of Pluripotent Stem Cells

**DOI:** 10.1038/srep31063

**Published:** 2016-08-10

**Authors:** Hiroki Sato, Alimjan Idiris, Tatsuaki Miwa, Hiromichi Kumagai

**Affiliations:** 1 Kumagai Fellow Laboratory, Innovative Technology Research Center, Technology General Division, Asahi Glass Co., Ltd., 1150 Hazawa-cho, Kanagawa-ku, Yokohama-shi, Kanagawa 221-8755, Japan

## Abstract

Various scalable three-dimensional culture systems for regenerative medicine using human induced pluripotent stem cells (hiPSCs) have been developed to date. However, stable production of hiPSCs with homogeneous qualities still remains a challenge. Here, we describe a novel and simple embryoid body (EB) formation system using unique microfabricated culture vessels. Furthermore, this culture system is useful for high throughput EB formation and rapid generation of differentiated cells such as neural stem cells (NSCs) from hiPSCs. The period of NSC differentiation was significantly shortened under high EB density culture conditions. Simultaneous mass production of a pure population of NSCs was possible within 4 days. These results indicate that the novel culture system might not only become a unique tool to obtain new insights into developmental biology based on human stem cells, but also provide an important tractable platform for efficient and stable production of NSCs for clinical applications.

Human pluripotent stem cells (hPSCs), including human embryonic stem cells (hESCs) and human induced pluripotent stem cells (hiPSCs), are capable of differentiating into the numerous cell types constituting all three embryonic germ layers^1^. Therefore, they are promising as source materials for treating various disorders^2^. For example, hPSC-derived neural stem cells (NSCs) and further differentiated neurons and glial cells have potential applications in biomedical sciences, such as modeling neurological disorders using disease-specific hiPSCs^3^, cell replacement therapies for refractory neuronal diseases^4^,^5^, and pharmacological and toxicological screening^6^,^7^. However, there are still two major challenges regarding cell culture processes to realize the therapeutic potential of hPSC derivatives, namely large-scale mass production and stable supply of cells with uniform quality.

Recently, various methods have been reported for scalable three-dimensional (3D) culture of hPSCs as cell aggregates or embryoid bodies (EBs) such as bioreactors^8^,^9^, functional polymers^10^,^11^, and microwell arrays^12^,^13^. Among these methods, the advantages of bioreactor culture systems include easy scale-up, controllable culture parameters, and labor cost efficiency^8^. However, stirring/agitation is often required to adjust for maintenance of the cell aggregation quality, because the appropriate conditions depend highly on the structural design of the bioreactor^14^. Despite extensive efforts, transplantation of differentiated cell aggregates produced in a bioreactor has not led an obvious influence on tissue repair processes^15^. Moreover, it has been reported that undifferentiated cells remain on peripheral cell aggregates with the unintended risk of tumor formation. Methods of suspension culture using functional polymers have been reported to enable long term expansion of hPSCs with high pluripotency, even with single cell seeding^11^. In particular, a culture system with a hydrogel containing a thermo-reversible polymer has enabled differentiation of dopaminergic progenitor cells from undifferentiated cell aggregates^11^. However, single cell culture enables reproducible expansion and EB formation that often require a long time to reach an appropriate size for effective differentiation. As described above, there are still some issues and limitations in current 3D suspension culture systems. Although many studies have implied that EB size affects stem cell differentiation processes^16^,^17^,^18^, the effect of EB size differences is poorly understood. The lack of research concerning such an effect is due, in part, to the difficulty inherent to quantitative creation of homogeneously sized EBs.

To overcome the abovementioned problems, we developed a novel culture method using unique culture vessels that allow rapid and mass production of homogeneous EBs with a controlled size. Unlike current 3D culture systems, our novel culture system is characterized by easy cultivation and EB formation of hiPSCs at a high cell density using microfabricated plastic dishes with flexible microwells.

In this study, we introduce experimental procedures for well-defined and efficient EB formation and expansion methods for hiPSCs. Then, we describe a new insight, which was revealed by application of the culture system, into the effect of EB size on the efficiency of neural lineage differentiation. We finally demonstrate an optimized protocol for the generation of a large number of NSCs under xeno-free culture conditions required for medical use. Overall, the results of the present study suggest that our culture systems are applicable to multiple uses of rapid and highly efficient EB formation and differentiation, and might provide an important and versatile technology platform for clinical and industrial purposes in the future.

## Results

### Formation of uniformly sized EBs using microfabricated culture vessels

**Figure 1:**
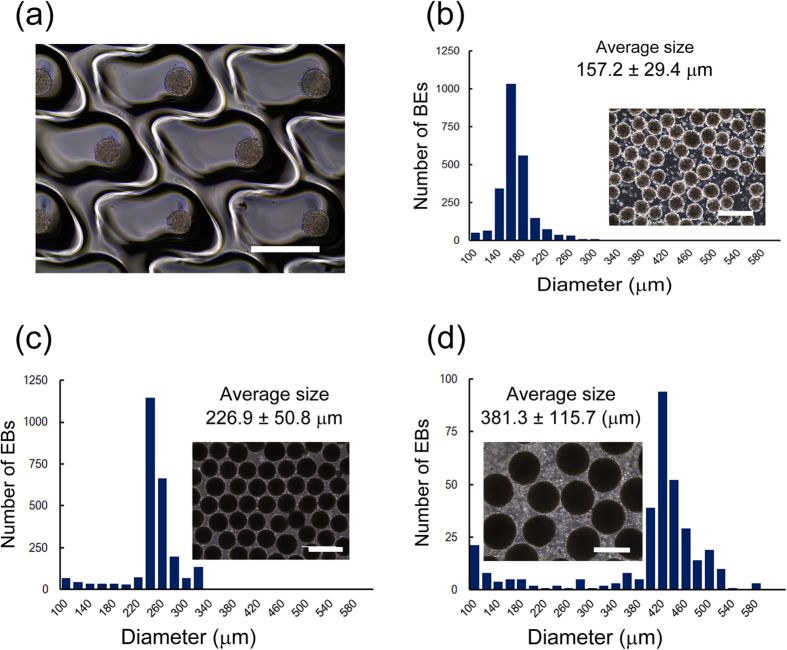
EB formation on the EZSPHERE with uniform and controlled sizes. (**a**) Representative microscopic image of EBs formed in the microwells of the EZSPHERE. (**b**–**d**) Comparison of the size distribution of the EBs formed at different seeding cell densities (**b**,**c**) or in different microwell sizes of the EZSPHERE (**d**) with maintenance medium containing Y-27632. EB size was analyzed by setting the cell seeding density as 400 cells (**b**) or 2,000 cells (**c**) per microwell using the same standard type EZSPHERE #900 (well diameter : depth = 500 μm : 100 μm), or a cell seeding density of 9,000 cells per microwell for the larger microwell type EZSPHERE #905 (well diameter : depth = 1,400 μm : 600 μm) (**d**). The resultant EB sizes were uniform and could be arranged as small (**b**), medium (**c**), and large (**d**). Scale bars: 400 μm. The differences in the size of EBs among each condition were statistically significant (p < 0.0001).

To establish a novel high throughput method for uniformly sized EB formation of hiPSCs with easy handling and high efficiency, we applied a unique type of microfabricated culture vessel, EZSPHERE, which is designed with a controlled uniform size of microwells on plastic dishes by laser-based microfabrication (Supplementary Figs 1 and 2a,b). When precultured and dissociated hiPSCs were seeded into the standard type of EZSPHERE (#900, microwell size: 500 μm in diameter and 100 μm in depth) at 400 cells per microwell, the cells spontaneously dropped into each microwell and promptly formed homogeneous EBs within 3–4 h (Fig. 1a,b and Supplementary Video). In contrast, static suspension culture onto a low-adhesion dish without microfabrication scarcely formed EBs within the same time (data not shown). We were able to obtain 2,378 EBs on a 35-mm dish-type EZSPHERE, which has approximately 2,400 microwells, indicating a high probability for EB formation. Live/dead-staining assay analysis of the obtained EBs revealed high cell viability (Supplementary Fig. 2c). The diametric size of the EBs was identified by the digital image analyzing software Image J, which showed a tight Gaussian distribution (157.2 ± 29.4 μm), indicating foremost size uniformity (Fig. 1b). Such uniformity was also observed using the 96-well plate-type EZSPHERE (Supplementary Fig. 2d). Using these vessels, we examined the relationship between the input cell number and the diameter of the formed EBs. As a result, the EB size (diameter) was correlated with the cell seeding numbers from 200 to 1,000 cells per microwell (Supplementary Fig. 2e).

To evaluate the size uniformity of EBs formed in different pore sizes of the created microwells, we compared the standard type EZSPHERE #900 with the EZSPHERE #905, which has a larger microwell pore size (1,400 μm in diameter and 600 μm in depth) (Supplementary Fig. 1). Seeding hiPSCs at a density of 2,000 cells per microwell on the standard type EZSPHERE (#900) resulted in the formation of midsize (226.9 ± 50.8 μm) EBs (Fig. 1c), whereas seeding at a higher density of 9,000 cells per microwell on the EZSPHERE (#905) resulted a larger EB size (381.3 ± 115.7 μm) (Fig. 1d). These data indicate that this novel and simple EB formation system enables easy and precise control of EB size by changing the cell seeding density and/or microwell size.

### Expansion culture of cell aggregates with pluripotency

**Figure 2:**
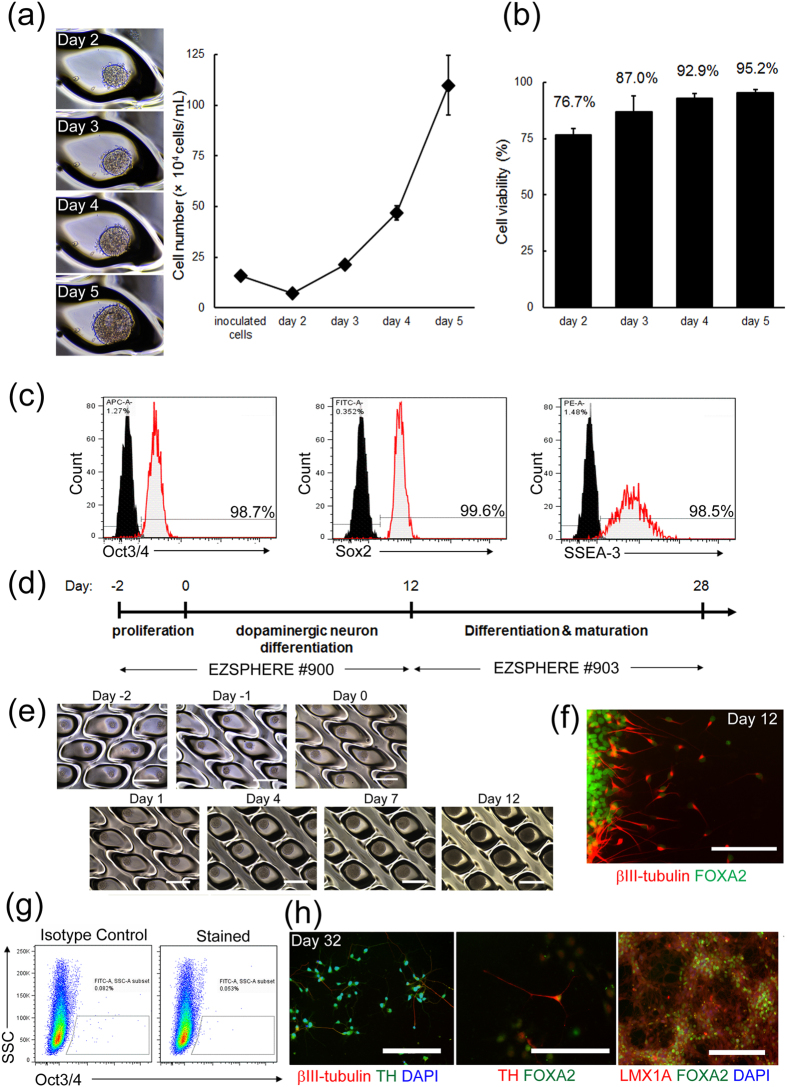
Efficient cell growth while maintaining high pluripotency on the EZSPHERE. (**a**) Representative microscopic images of fixed-point continuous observation of an EB formed in a microwell of an EZSPHERE #900. Progressive and continuous cell growth was observed during the time course of 5 days, and a cell growth curve was created by counting average cell numbers at each day in three independent experiments (n = 3). Scale bar: 200 μm. (**b**) Graph of the cell viability ratio assayed by trypan blue staining of the distributed cells from EBs. Each point represents the mean ± SD (bar) of triplicate. (**c**) Flow cytometric analysis of three major pluripotency markers, Oct3/4, Sox2, and SSEA-3, in cells obtained from EBs at day 5. The percentages of marker-positive populations are indicated in each panel, suggesting high level maintenance of pluripotency. (**d**) Schematic outline of the optimized protocol for induction of midbrain dopaminergic neurons on the EZSPHERE. (**e**) Phase-contrast images of a time course of 12 days of EB culture on the EZSPHERE. Scale bars: 400 μm (**f**). Fluorescence microscopy of immunostaining for the midbrain progenitor marker FoxA2 and neural marker βIII-tubulin at day 12. Scale bar: 200 μm. (**g**) Flow cytometric analysis for detection of undifferentiated cells (Oct3/4^+^) after the differentiation process at day 12. (**h**) Fluorescence microscopy of immunostaining for midbrain dopaminergic neuronal markers βIII-tubulin, TH, FoxA2, and LMX1A at day 32. Scale bars: 200 μm.

The existing protocols for EB formation of dissociated hPSCs using microwell plates or arrays are almost always used for limited purposes such as the EB formation step or early stage of differentiation^19^,^20^. To examine whether the EZSPHERE culture system could be applied to other processes, such as expansion and differentiation, we tested the proliferation ability of cells in EBs formed on the EZSPHERE. The initial cell seeding density was adjusted to 200 cells/microwell of the EZSPHERE (standard type), and the cells were cultured with feeder-free cell culture medium (mTeSR1) for 5 days. Microscopy revealed that the initially formed EBs at day 1 in each microwell had expanded and grew as cell aggregates, and the total cell number was increased by 15-fold from day 2 to 5 (Fig. 2a). Trypan blue staining indicated that the cells at day 5 were 95% viable (Fig. 2b).

Furthermore, flow cytometric analysis showed that >98% of the cells were positive for pluripotency markers Oct3/4, Sox2, and stage-specific embryonic antigen (SSEA)-3 (Fig. 2c). The maintenance of Oct3/4 and Sox2 expression levels during the EB formation and cultivation process was also confirmed by qRT-PCR analysis (data not shown).

### Continuous culture from proliferation to differentiation

To further evaluate the pluripotency of the obtained EBs, we examined neural lineage differentiation (Fig. 2d). For the differentiation process, soluble factors and small molecules, including SMAD inhibitors, a Sonic hedgehog (Shh) agonist, glycogen synthase kinase 3β (GSK3β) inhibitor, and fibroblast growth factor (FGF)-8, were sequentially added to the culture medium through medium changes after the initial expansion step with mTeSR1 medium. Cell proliferation in EBs under differentiation conditions was observed until day 12 (Fig. 2e), and differentiation into midbrain neurons, which are positive for markers forkhead box protein A2 (FoxA2) and βIII-tubulin, was confirmed (Fig. 2f). Flow cytometric analysis revealed that the ratio of Oct3/4-positive cells after the neural differentiation process was similar to the sample treated with isotype IgG, suggesting the absence of undifferentiated cells (Fig. 2g). At day 32, the obtained cells finally differentiated into midbrain dopaminergic neurons that are characterized as positive for βIII-tubulin, tyrosine hydroxylase (TH), LIM homeobox transcription factor 1, alpha (LMX1A), and FoxA2 (Fig. 2h). Most cells expressed postmitotic neuron marker, neuronal nuclei (NeuN). In contrast, there were little positive cells for the proliferation marker, Ki67 (Supplementary Fig. 3a). Moreover, potassium-evoked dopamine release was also confirmed (Supplementary Fig. 3b). These results clearly indicated that the created EBs on the EZSZPHERE have efficiently differentiated into the postmitotic and functional dopaminergic neurons in finally. Taken together, obtained EBs on the EZSPHERE could efficiently differentiate into lineage-specific cells, indicating that the novel culture system enables the culture of EBs for both cell expansion and differentiation processes on the same culture ware.

### Neural lineage differentiation in the novel culture system

**Figure 3:**
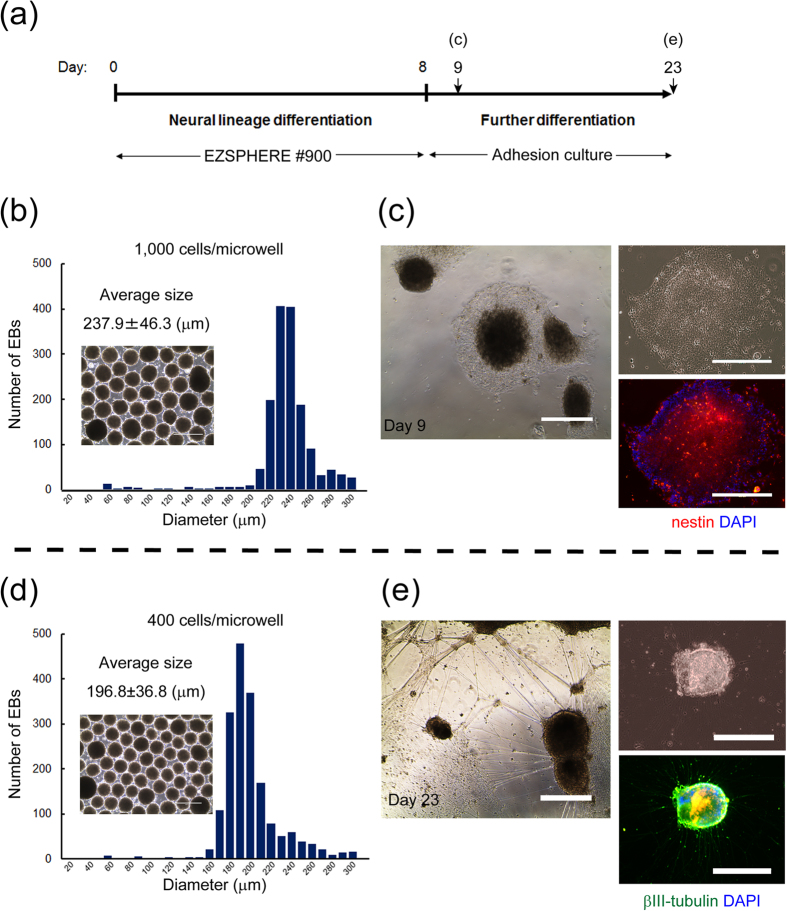
EB size dependence for neural differentiation fate. (**a**) Schematic outline of the procedure used to differentiate hiPSCs to neural lineage cells. EB generation and differentiation processes were performed on the EZSPHERE #900 for 8 days, followed by adhesion culture on Matrigel-coated glass bottom chambers. (**b**,**d**) Phase-contrast images of the obtained EBs and their size distributions. These EBs were generated by seeding precultured and dissociated hiPSCs at cell densities of 1,000 cells (**b**) or 400 cells (**d**) per microwell. (**c**) Phase-contrast and immunofluorescence images of the neural differentiated and extended EBs at day 9. Immunofluorescence staining was performed for the major neural stem cell marker nestin. (**e**) Phase-contrast and immunofluorescence images of the neural differentiated EBs at day 23. Immunofluorescence staining was performed for the neuronal marker βIII-tubulin. Scale bars: 400 μm.

Because biomedical and clinical applications of hPSCs and EBs require large scale cell expansion and differentiation procedures, we examined whether our culture system is suitable for the preparation of a large number of differentiated cells using neural differentiation processes as a model. Kondo *et al.* has reported a protocol for differentiating hiPSCs into neural progenitor cells using a spheroid plate (9,000 cells/well) with small molecular SMAD inhibitors^21^. To apply this differentiation protocol to our present system, hiPSCs were seeded into two EZSPHERE dishes at seeding densities of 400 and 1,000 cells per microwell, respectively, and differentiated into neural lineage cells by adding small molecule SMAD inhibitors. At day 8 after seeding, uniformly sized EBs were obtained with diametric sizes of 196.8 ± 36.8 μm and 237.9 ± 46.3 μm at low and high seeding densities, respectively, in the EZSPHERE dishes (Fig. 3b,d). Then, the obtained EBs were transferred onto Matrigel-coated glass-bottom chambers for further differentiation. The next day, large numbers of neural precursor/stem (nestin-positive) cells (NSCs) were observed as cells migrating from the edge of adhered EBs that were initially seeded at the higher cell density, but not the lower cell density (Fig. 3a, data not shown for the lower cell density). This result might suggest the importance of the initial cell seeding density or EB size for the induction efficiency of neural lineage cells. On the other hand, at the lower cell seeding density, attached EBs formed numerous cell protrusions as βIII-tubulin-positive neurites by 23 days of culture (Fig. 3b).

### Relationship of the cell seeding density and neural differentiation rate

**Figure 4:**
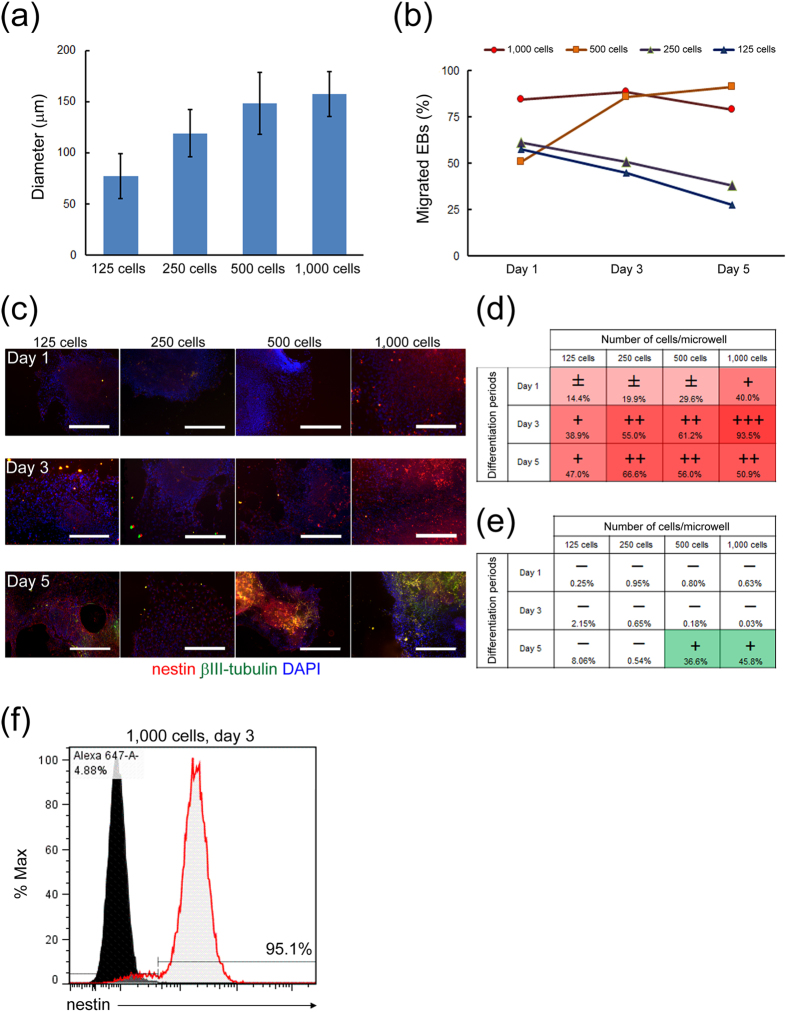
Cell seeding density-dependent acceleration of neural differentiation on the EZSPHERE. (**a**) Distribution of the EB size (diameter) resulting from initial cell seeding densities from 125 to 1,000 cells per microwell, followed by culture in neural differentiation medium for 3 days on the 96-well plate-type EZSPHERE #900. The initial seeded cell number in each microwell is indicated on the x-axis of the graph. The differences in the size of EBs among each condition were statistically significant (p < 0.0001). (**b**) Rational transition of the extended EB ratio for each EB size condition at days 1, 3 and 5 after transferring the differentiated EBs from the EZSPHERE onto Matrigel-coated chambers. A similar result was reproduced in a separate experiment. (**c**) Immunofluorescence microscopy for each type of differentiated EBs at days 1, 3 and 5, indicating the EB size- and cell seeding density-dependent shifts of the differentiation levels (nestin-positive neural stem/progenitor cells and βIII-tubulin-positive neurons were analyzed). (**d**,**e**) Quantitative analysis of the area stained positively for nestin (**d**) or βIII-tubulin (**e**) indicated in (**c**). The ratios were calculated using DAPI-stained cells as a reference. (**f**) Flow cytometric analysis of the neural stem cell marker nestin for the initial cell seeding density of 1,000 cells per microwell and neural induction for 3 days. The percentage of the marker-positive population is indicated in the panel. Scale bars: 400 μm.

The above results of neural differentiation suggested the possibility that the cell seeding density for EB formation or the resultant EB size on the EZSPHERE significantly affect the neural differentiation rate. To confirm such a possibility, further neural lineage differentiation was performed by varying the initial cell seeding density from 125 to 1,000 cells per microwell. As estimated, the resultant EB sizes were dependent on the initial cell seeding density (Fig. 4a). The obtained EBs were retrieved over time and immunofluorescence staining was performed to detect nestin and βIII-tubulin to track the differentiation process. After transferring the EBs from the EZSPHERE to adherent conditions, cells that migrated from EBs were observed after 1 day of differentiation induction and their ratio increased according to the EB size (Fig. 4b, day 1). As shown in Fig. 4b, at day 3 or later, cell-migrating EBs were observed at a high frequency (>75%) for larger EBs that were created by initially seeding 500 or 1,000 cells per microwell, but at a lower frequency for smaller EBs (Fig. 4b, day 3 and 5). Immunofluorescence staining of the differentiated cells at days 3 and 5 revealed that nestin was highly expressed in migrating cells from larger EBs at the early stage of the differentiation process (Fig. 4c). By quantifying the immunofluorescence intensity, we found that the highest ratio of 93.5% nestin-positive cells was obtained at day 3 for the initial seeding density of 1,000 cells/microwell (Fig. 4d). A similar result was confirmed in flow cytometric analysis (Fig. 4f). EB size and the time dependence of neural differentiation efficiency were also observed by detection of βIII-tubulin expression at day 5, which showed 35–45% positivity for larger EBs, but not smaller EBs (Fig. 4c,e). These results suggested that our culture system could shorten the period of differentiation into neural cells by high cell density culture of homogeneous EBs on the EZSPHERE. The obtained neural progenitor/stem cells were confirmed to differentiate further into neurons and astrocytes, suggesting their multipotency (Supplementary Fig. 3).

### Effect on each neural differentiation step

**Figure 5:**
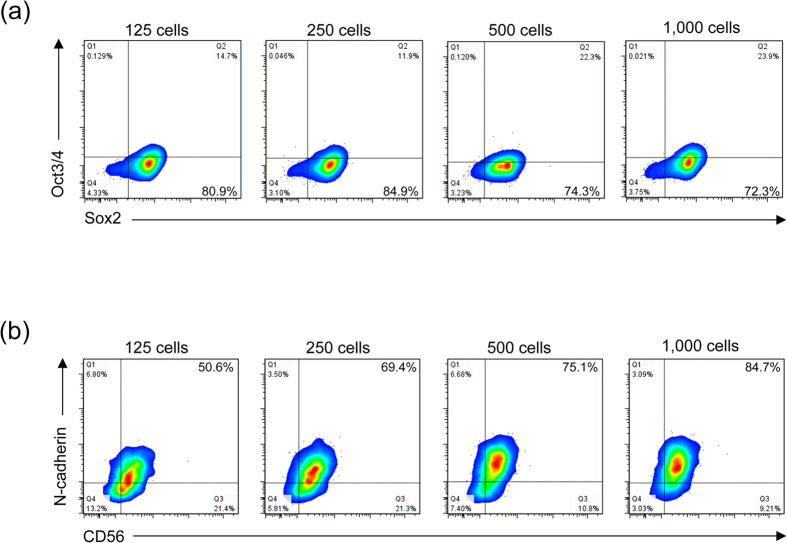
Flow cytometric analysis of neural differentiation of EBs formed at different cell seeding densities on the EZSPHERE. Flow cytometric analysis for detection of neuroepithelial cells (Oct3/4^−^/Sox2^+^) (**a**) and neural stem/progenitor cells (N-cadherin^+^/CD56^+^) (**b**) after induction of neural differentiation for 4 days. The percentage of each marker-positive population is indicated in each panel. The initial cell seeding density (cells per microwell) used to generate each type of EBs on the EZSPHERE is indicated at top of each panel.

To further investigate the shortening effect on the neural differentiation process in detail, we determined which step in the overall differentiation process was affected by the cell seeding density and/or EB size. The analysis was conducted by detecting the intensity of stage- and lineage-specific markers in cells during the neural differentiation process, which were initially seeded at different densities on the EZSPHERE. After 4 days of differentiation for each seeding condition, there was little difference in the ratio of neuroepithelial cells defined by detecting the ratio of Oct3/4^−^ and Sox2^+^ cells^22^,^23^ (Fig. 5a). Furthermore, a similar result was obtained by qRT-PCR analysis of the transcription levels of these markers (data not shown). In contrast, the ratio of neural progenitor/stem cells (NSCs) characterized as positive for CD56^24^ and N-cadherin^25^ improved according to the increase in the cell seeding density (Fig. 5b). This finding indicated that high cell density cultivation of hiPSCs as EBs accelerates the NSC differentiation step, but has almost no effect on the earlier neuroepithelial differentiation process.

### High throughput preparation of NSCs

**Figure 6:**
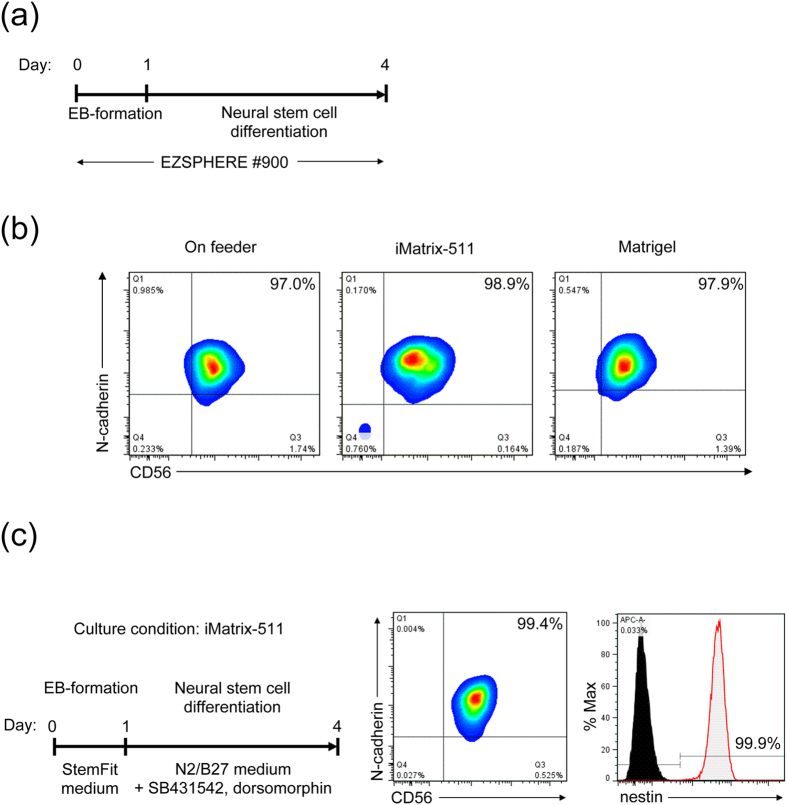
Stable and highly efficient NSC generation under different culture conditions. (**a**) Schematic outline of the optimized procedure for rapid and efficient induction of NSCs from hiPSCs on the EZSPHERE. (**b**) Flow cytometric analysis of the obtained NSCs induced from different EBs prepared by preculturing hiPSCs on distinct culture substrates of feeder cells, iMatrix-511, or Matrigel. (**c**) Preparation of NSCs under xeno-free culture conditions. The maintenance medium (StemFit AK02N) was used for preculture and EB generation. N2/B27 medium containing SB-431542 and dorsomorphin was used for neural differentiation. Analysis of neural stem cell markers, CD56, N-cadherin and nestin by flow cytometric analysis at day 4 of the differentiation. The percentage of the marker-positive population is indicated in each panel.

To confirm the appropriateness and validity of the present neural differentiation method for different culture conditions, we next evaluated the NSC generation efficiency by changing the maintenance conditions including the use of feeder cells or artificial matrices such as iMatrix-511, a recombinant product of C-terminal fragment of the laminin-511, and Matrigel. After preparation of hiPSCs using the above-described conditions, followed by 1 day of EB formation in each maintenance medium containing Y-27632, differentiation induction into neural lineage cells with differentiation medium containing small molecule SMAD inhibitor was performed for 3 days. The obtained EBs were then dissociated and analyzed by flow cytometry. The results showed that >97% of cells had effectively differentiated into NSCs (Fig. 6a,b). This result indicates that, even using different extracellular matrices or scaffolds before seeding into the EZSPHERE, it is possible to obtain very high efficiency of NSC induction within 4 days using the present system. Furthermore, to evaluate whether this protocol can be adapted to xeno-free conditions, which are required for most clinical applications, differentiation of hiPSCs into NSCs was attempted by culture on iMatrix-511 using the commercial medium StemFit AK02N, a chemically defined and animal component-free medium that was recently developed by Ajinomoto. After EB formation on the EZSPHERE for 1 day, the StemFit AK02N medium was changed to N2B27 medium for differentiation. Similar to the results described above, flow cytometric analysis indicated that >99% of cells had efficiently differentiated into NSCs positive for nestin, CD56, and N-cadherin (Fig. 6c).

## Discussion

Development of large scale production and processing with low costs and reproducible culture techniques for PSCs, including hESCs and hiPSCs, will be essential for the practical applications of regenerative medicine and drug discovery. Accordingly, various 3D suspension culture systems for expansion and differentiation of hPSCs have been reported to date^8^,^10^,^11^,^26^.

Conversely, because various types of differentiated cells are induced from hPSCs, further development of key technologies is also required for rapid preparation of cells with low costs and highly versatile states such as EBs or NSC spheroids. EB formation is a general and common step in the process of producing distinct types of differentiated cells from hPSCs. In addition, EB size is known to affect the differentiation efficiency and rate^17^,^18^. Therefore, development and optimization of EB formation methods are very important. Among the types of multipotent stem cells, it has been shown that NSCs derived from PSCs can differentiate into neuron, astrocyte, and oligodendrocyte lineages *in vitro*^27^. These cells are highly anticipated as a cell source for drug discovery to treat congenital neural disorders as well as regeneration of neural tissues lost as a result of neurodegenerative diseases and injuries^28^.

In general, it is known that the size uniformity and productivity of stem cell aggregates are a trade-off relationship^29^. In this study, we overcame such problems by establishing a novel reproducible protocol for preparation of a large amount of uniformly sized EBs using the unique microfabricated culture vessel EZSPHERE (Figs 1 and 2). Moreover, using an optimized and well-defined culture system, we found that the initial cell seeding density greatly affects the neural differentiation rate of hiPSCs (Figs 3, 4, 5). Finally, we succeeded at significantly shorting the total time for NSC differentiation from EBs on the EZSPHERE by optimization of the culture conditions (Fig. 6). This protocol was more satisfactory than other microfabric vessels such as AggreWell (StemCell Technologies) (data not shown).

Inhibition of SMAD signaling is a powerful technique to induce the differentiation of PSCs into neural cells. Chambers and colleagues reported a protocol using SMAD inhibitors, Noggin, and SB-431542, in 2009. hESCs and hiPSCs can be differentiated into PAX-6 (neural precursor cell marker)-positive cells in 11 days at a high differentiation rate (80%) with this protocol^30^. With a recently developed protocol using the low molecular weight compound dorsomorphin in place of Noggin, 99.6% of cells underwent differentiation into PSA-NCAM (CD56) (neural stem cell marker)-positive cells in 14 days^31^. Meanwhile, Ashton and colleagues reported that hPSCs differentiated into pure neuroepithelial cells within 6 days under adherent conditions^32^. Our differentiation induction protocol is significantly superior to the previously reported protocols because almost all cells differentiate into CD56- or N-cadherin-positive and nestin-positive NSCs in a short time (4 days; essentially 3 days excluding the time required for EB formation), despite the same concept used for its development. Nestin has been unequivocally accepted as a marker of NSCs both during embryonic development and in the adult brain. In addition, it is reported that nestin has an important function in the survival and proper self-renewal of neural stem cells^33^. To the best of our knowledge, the period required for induction of NSC differentiation demonstrated in the present study is the shortest among the previously reported methods. In addition, cells with uniform characteristics can be produced with any culture lot using our novel experimental protocol, because NSCs are producible at a high rate even in xeno-free, chemically defined culture media. Furthermore, the NSCs generated with the present protocol possess a high capacity for further differentiation into various neural cell types such as neurons and glial cells (Supplementary Fig. 3). Therefore, such NSCs can be used for cell transplantation to treat progressive neurological disorders such as Parkinson’s disease and amyotrophic lateral sclerosis. Our short term neuronal differentiation induction protocol will be a major step toward achieving autotransplantation without immunorejection based on the most important feature of iPSCs in clinical settings. The present study has demonstrated for the first time significant shortening of the differentiation time using a microfabricated culture vessel such as the EZSPHERE.

Because our present culture system is distinguishable by adopting a unique method of cultivation and EB formation of hPSCs at a high cell density, we hypothesized that cellular interactions, especially the effects of endogenous factors secreted from EBs, in the culture system more effectively influence cells cultured on the EZSPHERE than in other 3D culture systems. Previous studies have reported that hESCs constantly express and secrete FGF-2 that is important to maintain the undifferentiated state during stem cell cultivation^34^. In our culture system, high density EB culture might be one of the reasons for the proliferation of hiPSCs with an undifferentiated state, despite the simple culture conditions lacking cell-extracellular matrix interactions that are known to play an important role in controlling various stem cell properties (Fig. 2). In the neuronal differentiation process, it has been reported that endogenous FGF-4 and other factors activate downstream signaling to promote differentiation of the primitive ectoderm (neuroepithelium) into neural precursor cells in hESC-derived EBs^22^,^24^,^35^. Additionally, during embryonic development *in vivo*, intrinsic FGF-8 is known to be a strong neural differentiation inducer that induces homogenous differentiation into neural lineage cells^22^,^36^. These previous reports might support our experimental results showing that improved efficiency of differentiation into neural precursor/stem cells is dependent on the cell seeding density (Fig. 5). Because we are planning to analyze the components of the culture supernatant, future investigation might elucidate the mechanistic contributions of the above advantageous features of the present culture system.

It has been also reported that a large EB size (approximately 500 μm in diameter) restricts the expression of initial neuronal tissue markers such as Hes1 and βIII-tubulin^37^. Based on the above knowledge, our experimental system for cultivation of small EBs at a high density on microfabricated culture vessels may be suitable for effective and rapid preparation of NSCs. The most important point in this study is that the novel culture system has provided a new insight into stem cell biology as described above.

EB size also affects the differentiation rate of not only neural lineage cells, but also cardiac and endothelial cells^18^. Moreover, it has been reported that induction of cardiomyocyte differentiation from EBs cultured at a high density increases the effects of endogenous bone morphogenetic proteins (BMPs) and markedly promotes carcinogenesis and the production of greater quantities of functional cardiomyocytes that express typical cardiac markers Mlc2v, NKX2.5, and cTnT^38^. Therefore, the present EZSPHERE culture system also has a high potential to provide more optimal conditions for generating cardiomyocytes from hPSCs and might become a powerful tool for cardiac differentiation as well as for neural differentiation. To demonstrate such a viewpoint, a study of cardiac differentiation of hPSCs in our system is currently ongoing.

The EBs and NSC spheroids generated in our system can be further expanded or processed at a larger scale using bioreactors. In addition, the EZSPHERE is microfabricated to normal cell culture vessels with a size conforming to the SLAS (Society for Laboratory Automation and Screening) standard. Recently, there have been many reports about the establishment of automated and robotic processes for the maintenance and differentiation of hiPSCs^39^,^40^. There is the possibility to further improve the present protocol by combination with these automation technologies.

The significance of this study lies in not only efficient EB formation and neural differentiation, but also the establishment of key technologies that may provide a mass culture platform for hPSCs to realize several great potentials. This experimental system is theoretically a culture technique for potentiating the mechanism(s) by which hPSCs form, autonomically maintaining the undifferentiated state, and enhancing differentiation. Our culture system is thus anticipated to be a milestone in hPSC culture.

## Methods

### iPSCs, media, and culture conditions

The hiPSC line 201B7 provided by iPS Academia Japan, Inc. was used in all experiments. For conventional adherent culture, 201B7 cells were cultured on mitomycin C-treated SNL cells in Primate ES Cell Medium (ReproCELL, Yokohama, Japan) supplemented with 4 ng/mL FGF-2 (Wako Pure Chemicals Industries, Osaka, Japan). The medium was changed every day, and the cells were passaged using CTK (ReproCELL). For feeder-free adherent culture, hiPSCs were grown on Matrigel (Corning, NY) or iMatrix-511 (Nippi, Tokyo, Japan) with mTeSR1 medium (STEMCELL Technologies, Vancouver, Canada) or StemFit AK02N (Ajinomoto, Tokyo, Japan). Cultured cells were passaged by the cell dissociation enzyme Accutase (Sigma Aldrich, St. Louis, MO) or TrypLE Select (ThermoFisher Scientific, Waltham, MA) and then dissociated and seeded using maintenance medium containing 10 μM Y-27632 (Wako Pure Chemicals Industries). All cell culture was performed at 37 °C with 5% CO_2_.

### Cell growth assay

201B7 cells grown on Matrigel with mTeSR1 were resuspended in mTeSR1 supplemented with 10 μM Y-27632 and seeded into a 96-well plate-type EZSPHERE #900 (AGC Techno Glass, Shizuoka, Japan) at a cell density of 200 cells/microwell. The culture medium was changed at half volumes every day. Obtained cell aggregates as EBs were dissociated with 0.25% trypsin-EDTA (ThermoFisher Scientific) for cell counting and viability assays each day using an automated cell counter (TC10 Automated Cell Counter; BioRad, Hercules, CA).

### Live/dead assay of hiPSCs

Assessment of cell viability on the EZSPHERE was performed using a Live/Dead Cell Staining Kit II (Promokine, Heidelberg, Germany). At 24 h after cell seeding, the formed EBs were treated with 2 μM Calcein-AM and 4 μM EthD-III for 30 min at 37 °C. Stained cells were observed under a fluorescence microscope (EVOS FL auto; ThermoFisher Scientific).

### EB formation of hiPSCs

#### Using feeder-dependent hiPSCs

201B7 cells cultured on SNL cells were incubated with CTK for 1 min at 37 °C. Then, the cells were incubated with Accutase containing 50 μM Y-27632 for 5 min at 37 °C to form a single cell suspension. The obtained cell suspension was centrifuged at 190 × *g* for 3 min at 4 °C. The supernatant was removed and the cell pellet was resuspended in Primate ES Cell Medium supplemented with 5% KnockOut Serum Replacement (KSR) (ThermoFisher Scientific) and 50 μM Y-27632, 10 μM SB-431542 and 2 μM dorsomorphin (Wako Pure Chemicals Industries). The obtained cells were counted to determine the cell number and viability. Live cells were seeded at 9.2 × 10^5^ or 4.6 × 10^6^ cells into a 35-mm dish-type EZSPHERE #900 (approximately 400 and 2,000 cells/microwell) for 3 or 8 days, respectively. To form EBs with a larger size using an EZSPHERE #905 (approximately 9,000 cells/microwell), about 1.8 × 10^6^ dissociated hiPSCs were seeded into the EZSPHERE and incubated for 3 days. Half volumes of the culture medium were carefully replaced at days 1 and 4.

#### Using feeder-free hiPSCs

Cells cultured on Matrigel or iMatrix-511 with mTeSR1 medium or StemFit AK02N were treated with Accutase containing 10 μM Y-27632 or 0.5× TrypLE Select for 10 min at 37 °C, respectively. The dissociated cells were centrifuged at 300 × *g* for 5 min. The supernatant was removed and the cell pellet was resuspended in medium supplemented with 10 μM Y-27632, respectively, and then seeded into the EZSPHERE.

### Quantitative analysis of the EB size distribution

Diametrical sizes of the EBs were analyzed with Image J software (NIH; http://rsbweb.nih.gov/ij/) using images obtained under the fluorescence microscope (EVOS FL auto).

### Neural lineage differentiation of EBs

Cells precultured on feeder cells were treated with CTK for 1 min at 37 °C and then dissociated with Accutase containing 50 μM Y-27632 for 5 min at 37 °C. The dissociated cells were centrifuged at 190 × *g* for 3 min at 4 °C, and the pelleted cells were resuspended in Primate ES Cell medium supplemented with 50 μM Y-27632, 5% KSR, SB-431542, and dorsomorphin for neural differentiation. These cells were seeded into a 35-mm dish- or 96-well plate-type EZSPHERE #900 and cultured. Half volumes of the culture medium were carefully replaced at days 1 and 4. The obtained EBs were plated onto Matrigel-coated glass bottom chambers for induction of neural lineage differentiation. Each culture period for differentiation is described in Figs 3 and 4.

### Optimization of NSC differentiation induction

Cells precultured on different matrices or feeder conditions were harvested and suspended in each maintenance medium supplemented with Y-27632, and then seeded individually into a dish-type EZSPHERE #900 at a density as described in Fig. 5 or 1,000 cells/microwell as shown in Fig. 6. The next day, the culture medium of the generated EBs was changed to neural differentiation medium consisting of GMEM supplemented with 8% KSR, non-essential amino acids, sodium pyruvate, L-glutamine (all purchased from ThermoFisher Scientific), and SB-431542, followed by further cultivation for 3 days of differentiation.

### NSC induction under xeno-free culture conditions

Cells precultured on iMatrix-511 were dissociated with 0.5× TrypLE Select for 10 min at 37 °C. After washing twice with PBS (Wako Pure Chemicals Industries), the cells were resuspended and seeded into a 96-well type EZSPHERE #900 at a density of 8 × 10^4^ cells/well (approximately 1,000 cells/microwell) with xeno-free medium StemFit AK02N supplemented with 10 μM Y-27632. The next day, the culture medium of the generated EBs was replaced with N2B27 medium consisting of a 1:1 mixture of DMEM/F12 GlutaMAX and Neurobasal medium supplemented with 1× N2 supplement, 1× B27 supplement without vitamin A (all purchased from ThermoFisher Scientific), non-essential amino acids, sodium pyruvate, L-glutamine, SB-431542, and dorsomorphin. The cells were cultured for a further 3 days to induce NSCs.

### Dopaminergic neuron induction on the EZSPHERE

Dopaminergic neuron differentiation of EBs was performed on the 96-well type EZSPHERE #900 using previously reported medium conditions^41^. 201B7 cells precultured on iMatrix-511 with StemFit AK02N were dissociated with 0.5× TrypLE Select for 10 min at 37 °C and then rinsed with PBS. The dissociated cells were resuspended in StemFit AK02N supplemented with 10 μM Y-27632 and seeded into the 96-well type EZSPHERE #900 at a cell density of 1.6 × 10^4^ cells. Half volumes of the culture medium without Y-27632 were changed every day. After 2 days of cultivation, the medium was changed to differentiation medium (GMEM supplemented with 8% KSR, non-essential amino acids, sodium pyruvate, and L-glutamine) with LDN193189 (ReproCELL) and A-83-01 (Wako Pure Chemicals Industries) to induce neural differentiation. Purmorphamine (Cayman Chemical Company, Ann Arbor, MI) and FGF-8 (Wako Pure Chemicals Industries) were added from day 1 to 7, and CHIR99021 (Abcam, Cambridge, UK) was added from day 3 to 12. At day 12, the obtained EBs were dissociated by treatment with TrypLE Select for 10 min at 37 °C and rinsed with neural maturation medium consisting of Neuobasal medium supplemented with 1× B27 supplement, L-glutamine, 10 ng/ml glial cell line-derived neurotrophic factor (R&D Systems, Minneapolis, MN), 200 μM ascorbic acid (Sigma Aldrich), 20 ng/ml brain-derived neurotrophic factor (R&D Systems), and 400 μM dbcAMP (Sigma Aldrich). Suspended cells were seeded into a 35-mm dish type EZSPHERE #903 at a cell density of 2 × 10^6^ cells/dish (approximately 2,000 cells/microwell). The cells were then cultured for 28 days on the Matrigel-coated glass bottom chamber (8-well Chamber Slide II, AGC Techno Glass) to confirm differentiation into midbrain dopaminergic neurons.

### Neuronal and glial differentiation of NSCs

Induction of differentiation into neurons (motor neurons) or glial cells (astrocytes) was performed using a previously reported protocol^21^,^42^. For neuronal differentiation, the obtained NSC aggregates were cultured in DMEM/F12 GlutaMAX supplemented with 1 μM retinoic acid (Sigma Aldrich) and 1.5 μM purmorphamine for 5 days. Then, the cells were plated onto a Matrigel-coated glass-bottom chamber. For glial differentiation, NSCs were transferred onto a Matrigel-coated 6-well culture plate and cultured for 20 days in glial differentiation medium consisting of DMEM/F12 supplemented with 5% KSR, 1× N2 supplement, 10 ng/mL human BMP4 (R&D Systems), and 10 ng/mL human LIF (Merck Millipore, Darmstadt, Germany).

### Flow cytometry

201B7 cells or their differentiated cells were dissociated by treatment with TrypLE Select at 37 °C for 10 min. A portion of the dissociated cells was used to check their viability using a VivaFix Cell Viability Assay (BioRad, 1/500 in PBS), while the main portion of the cells was resuspended in 2 mL of 1% bovine serum albumin (BSA)/PBS and centrifuged for 5 min at 300 × *g*. The supernatant was removed and cell pellets were subjected to staining with fluorescently labeled antibodies for flow cytometric analysis.

For immunofluorescent staining of various cell surface markers with their corresponding fluorescently labeled antibodies, each cell pellet obtained above was resuspended in 50 μL of 1% BSA/PBS and 2 μL of the following fluorescently labeled antibodies: anti-CD56 antibody (BioLegend, San Diego, CA), anti-N-cadherin antibody (R&D Systems), and anti-CD44 antibody (BD Biosciences, Franklin Lakes, NJ). For immunofluorescence staining of intracellular markers, the cells were permeabilized with a transcription factor staining buffer kit Mouse/Human Pluripotent Stem Cell Multi-Color Flow Cytometry Kit (R&D Systems). Briefly, each cell pellet was resuspended at 5 × 10^5^ cells in 500 μL Fixation/Permeabilization Buffer and incubated at 4 °C for 30 min. The centrifuged cell pellets were resuspended again with 50 μL Permeabilization/Wash Buffer and 2 μL of the following fluorescently labeled antibodies: anti-Oct3/4 antibody, anti-Sox2 antibody, anti-SSEA-3 antibody (R&D Systems), anti-nestin antibody, anti-doublecortin antibody, or anti-glial fibrillary acidic protein (GFAP) antibody (BD Biosciences), and then incubated at 4 °C for 20 min. Finally, 2 mL Permeabilization/Wash Buffer or 1% BSA/PBS was added to the cell suspension, followed by centrifugation at 300 × *g* for 5 min at 4 °C. The obtained cell pellets were resuspended in 300 μL of the same buffer and subjected to flow cytometric analysis. A BD FACS Verse flow cytometer (BD Biosciences) was used for all flow cytometry. Raw data were analyzed using FLOWJO (Tomy Digital Biology Company, Tokyo, Japan).

### Immunofluorescence staining

Differentiated cells cultured onto a Matrigel-coated glass bottom chamber were fixed with 4% paraformaldehyde (Wako Pure Chemicals Industries) for 30 min at room temperature or with acetone⁄methanol (1:1, v/v) for 15 min on ice. The cells were then treated with a 0.5% Triton X-100 (Sigma Aldrich) solution for 15 min at room temperature to permeabilize the cells. The fixed cells were treated with 3% BSA/PBS for 1 h to block non-specific adsorption of antibodies and then incubated for a further 2 h at room temperature or overnight at 4 °C with primary antibodies against TH, βIII-tubulin, microtubule-associated protein-2 (MAP2) (Merck Millipore), GFAP (Dako, Glostrup, Denmark), FoxA2 (R&D Systems), Ki67 (BD Biosciences), NeuN (Merck Millipore) or LMX1A (Bioss antibodies, Woburn, MA). Each antibody was diluted with blocking buffer before use. After washing three times with PBS containing 0.1% Tween-20, the cells were treated for 1 h at room temperature with the corresponding fluorescently labeled secondary antibodies: fluorescein-labeled anti-mouse IgG (Vector Laboratories, Burlingame, CA), Alexa Fluor 594-labeled goat anti-mouse-IgG, Alexa Fluor-350-labeled goat anti-mouse IgG, or Alexa Fluor-488-labeled goat anti-rabbit IgG (ThermoFisher Scientific). Each secondary antibody was diluted at 1:200. The unbound antibodies were removed by washing three times with PBS containing 0.1% Tween-20. Cell nuclei were counterstained with a DAPI solution (Dojindo Laboratories, Kumamoto, Japan) according to the manufacturer’s protocol. Detection of the fluorescently labeled secondary antibodies was performed under the fluorescence microscope (EVOS FL auto).

### Real-time PCR

The total RNA was extracted with the PureLink RNA Mini Kit (ThermoFisher Scientific). Complementary DNA (cDNA) was prepared from RNA by reverse transcription using the High Capacity cDNA Reverse Transcription Kit (ThermoFisher Scientific). The reaction mixtures (20 μL), which contained the THUNDERBIRD SYBR qPCR Mix (TOYOBO, Osaka, Japan), cDNA (10 ng) and each primer (200 nM), were subjected to PCR using Stratagene Mx3000P (Agilent Technologies, Santa Clara, CA). The expression levels of each gene were normalized to the GAPDH expression. Relative expression levels of each marker gene were then deduced as the iPSCs in the maintenance culture conditions as base. The primer sequences used in the PCR were as follows: GAPDH forward primer, 5′-TGCACCACCAACTGCTTAGC-3′; GAPDH reverse primer, 5′-GGCATGGACTGTGGTCATGAG-3′; Oct3/4 forward primer, 5′-TCCCATGCATTCAAACTGAGG-3′; Oct3/4 reverse primer, 5′-CCTTTGTGTTCCCAATTCCTTCC-3′; Sox2 forward primer, 5′-GGGAAATGGGAGGGGTGCAAAAGAGG-3′; Sox2 reverse primer, 5′-TTGCGTGAGTGTGGATGGGATTGGTG-3′.

### Dopamine release assay

The obtained dopaminergic neurons (at day 28) were transferred onto the Matrigel-coated 96-well plates, then washed twice with a low KCl solution (phenol-free, Ca^2+^Mg^2+^-free Hank’s balanced salt solution (HBSS; Wako Pure Chemicals Industries)) and incubated for 15 min. The medium was subsequently replaced with a high KCl solution (56 mM) for 15 min. The solution was collected, and the concentration of dopamine was determined by a commercial dopamine ELISA kit (Abnova, Taipei, Taiwan).

## Additional Information

**How to cite this article**: Sato, H. *et al.* Microfabric Vessels for Embryoid Body Formation and Rapid Differentiation of Pluripotent Stem Cells. *Sci. Rep.* **6**, 31063; doi: 10.1038/srep31063 (2016).

## Supplementary Material

Supplementary Information

Supplementary Video
