# Supplementary material for: Microfabric Vessels for Embryoid Body Formation and Rapid Differentiation of Pluripotent Stem Cells
Source: Sci Rep. 2016 Aug 10;6:31063. doi: 10.1038/srep31063 (PMC4978968; doi:10.1038/srep31063)
Supplement: Supplementary Information [file srep31063-s1.doc]

**Supplementary Information**

**Microfabric Vessels for Embryoid Body Formation and Rapid Differentiation of Pluripotent Stem Cells**

**Hiroki Sato*, Alimjan Idiris*, Tatsuaki Miwa and Hiromichi Kumagai**

^1^Kumagai Fellow Laboratory, Technology General Division, Innovative Technology Research Center, Asahi Glass Co., Ltd., 1150 Hazawa-cho, Kanagawa-ku, Yokohama-shi, Kanagawa 221-8755, Japan

*Correspondence should be addressed to H.S. (n115353a@gmail.com) or A.I. (alimujiang-yidiresi@agc.com)

**
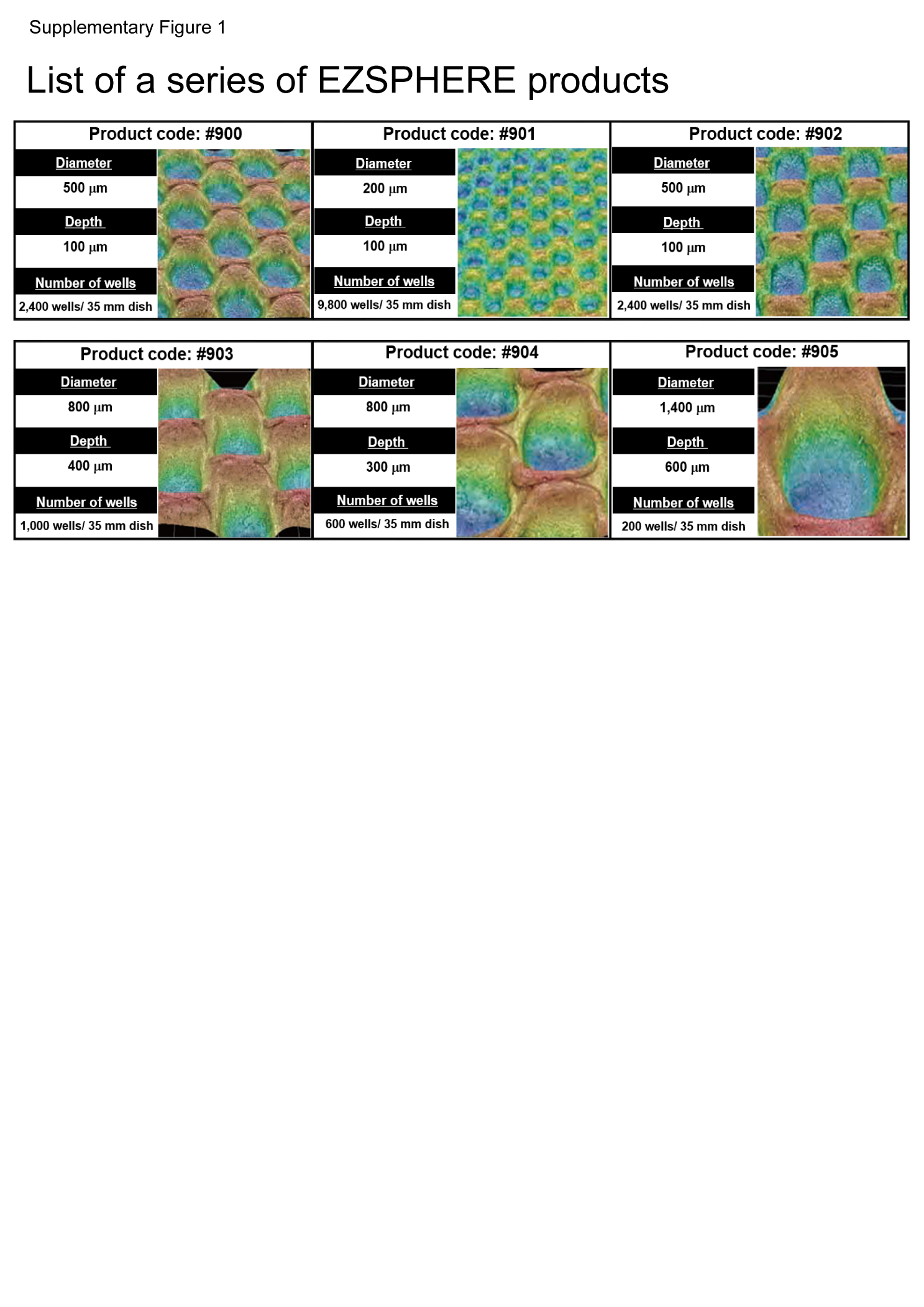
**

**Supplementary Fig. 1. EZSPHEREs designed with different microwell sizes**

**
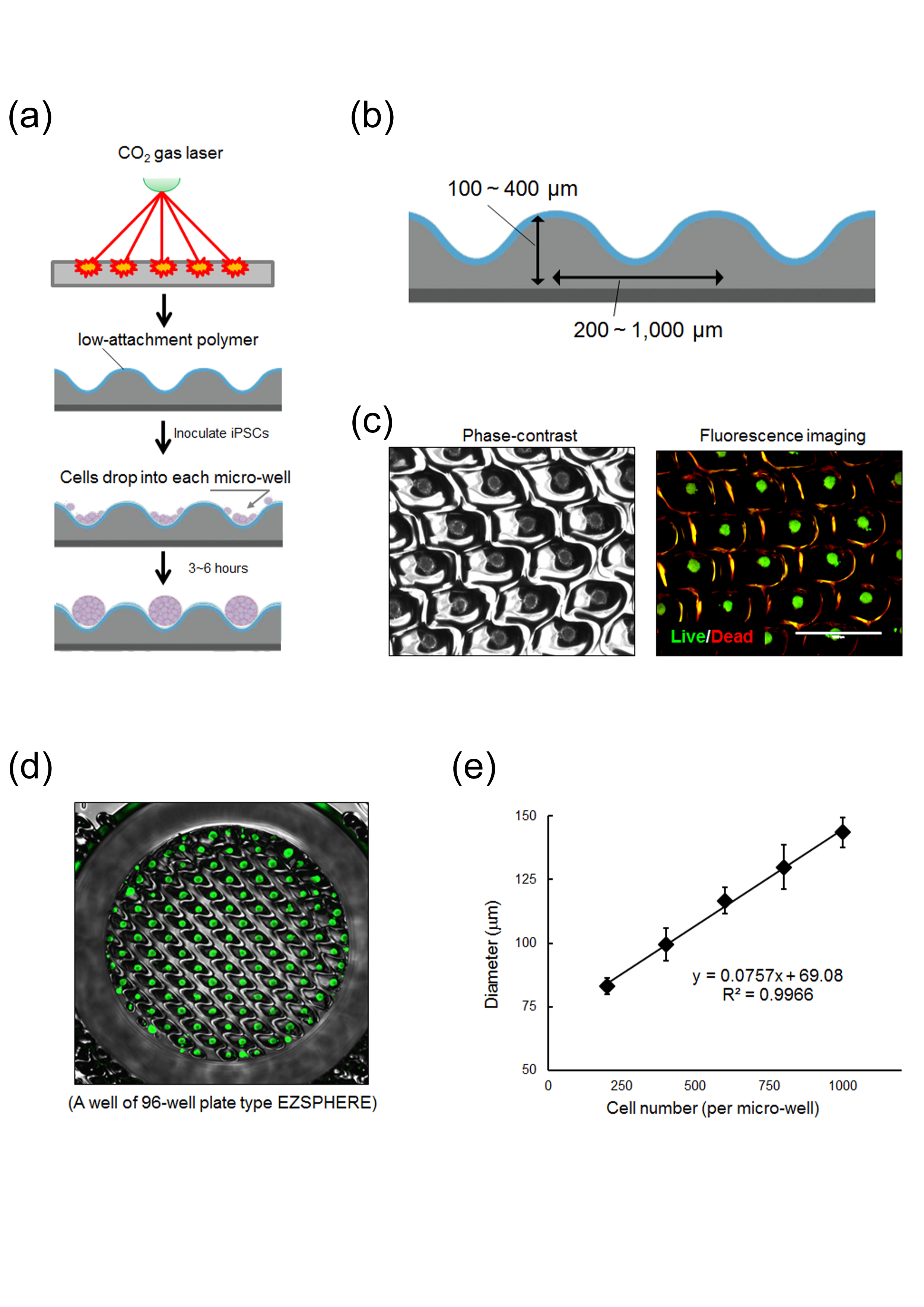
**

**Supplementary Fig. 2. EZSPHERE and its application for creating EBs with a uniform and controlled size.**

**a.** Illustrated scheme of microfabrication and EB formation processes of the EZSPHERE. The culture vessels are plastic culture dishes or plates with microwells created on the culture surface by a CO_2_ gas laser, followed by coating with low cell attachment and low protein-binding regents. The diameter and depth of each microwell can be easily altered to around 200–1,000 μm and 100–400 μm, respectively, by tuning the exposure time or intensity of the laser beam during the microfabrication process. Because the laser-processed surface is very smooth and the created microwells are closely positioned to each other (almost no flat areas occur between them), seeded cells are able to smoothly and equally drop into each well and contribute to EB formation. **b.** Illustrated sectional view of the created microwells on the EZSPHERE. **c.** Phase-contrast and live/dead-stained fluorescence micrographs of EBs generated in the microwells of a 35-mm dish type EZSPHERE #900. **d.** Live cell fluorescence micrograph of EBs generated in the microwells of a well of a 96-well plate type EZSPHERE. **e.** A good linear-dependence was found between the cell seeding number and EB size. The values are the mean ± SD. Scale bars: 1,000 μm.


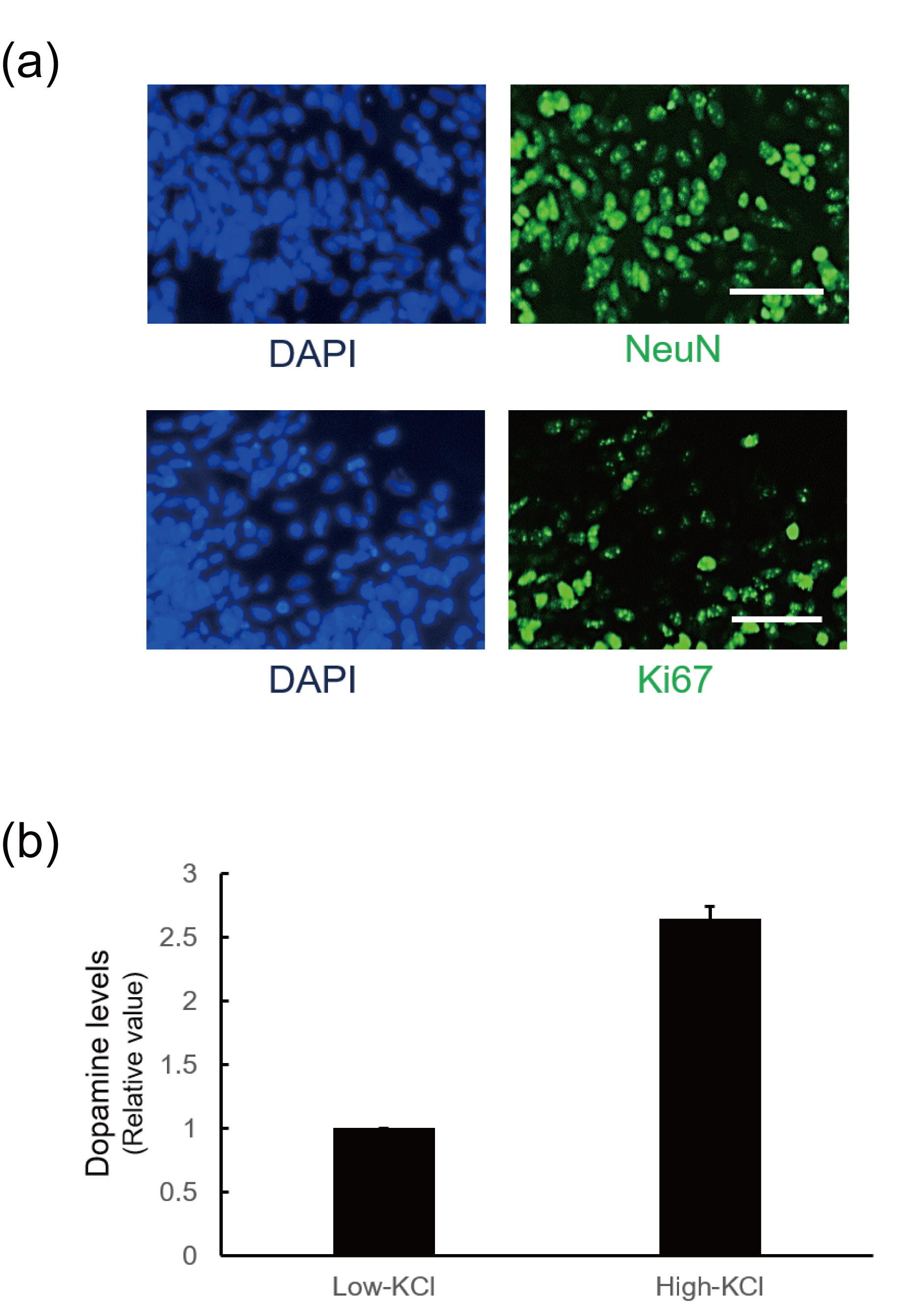


**Supplementary Fig. 3. Characterization of dopaminergic neurons**

**a.** Immunofluorescence images of the dopaminergic neurons for NeuN (post-mitotic neurons) or Ki67 (proliferating cells) (green) and DAPI (blue) at day 28. Scale bars: 200 μm. **b.** Detection of doparmine release level by EILSA assay. The values are the mean ± SD (bar).

**
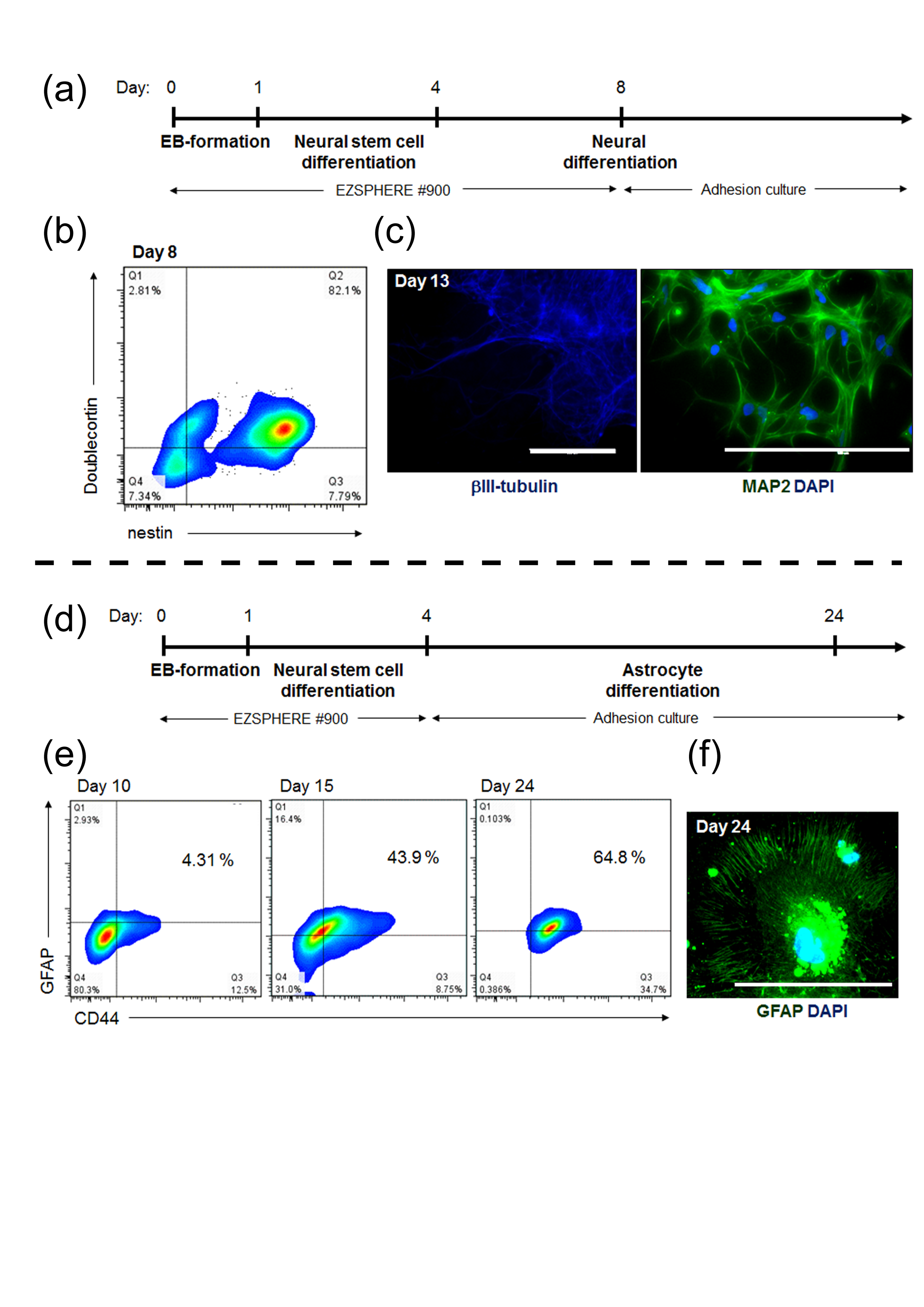
**

**Supplementary Fig. 4. NSCs further differentiate into neurons and glial cells**

**a.** Schematic outline of the optimized protocol for differentiation of neuronal cells from the obtained NSCs. **b.** Flow cytometric analysis for neural progenitor/neuronal cell markers nestin and doublecortin at day 8. **c.** Fluorescence microscopy of immunostaining for neural progenitor/neuronal cell markers βIII-tubulin and MAP2. **d.** Schematic outline of the optimized protocol for induction of glial cells (partially astrocytes) from the obtained NSCs. **e.** Flow cytometric analysis of astrocyte markers GFAP and CD44 at days 10, 15 and 24. **f.** Fluorescence microscopy of immunostaining for astrocyte marker GFAP at day 24. Scale bars: 200 μm.

**Supplementary Video**

Representative time-lapse video microscopy of the EB formation process on the EZSPHERE #900. hiPSCs seeded at a density of 1,000 cells/microwell spontaneously dropped into each microwell and promptly aggregated on the bottom as uniform EBs within 3–4 h. The video was captured continuously for 5 h by recording images every 12 min.
